# Using theory of change to develop an intervention theory for designing and evaluating behavior change SDApps for healthy eating and physical exercise: the OCAPREV theory

**DOI:** 10.1186/s12889-019-7828-4

**Published:** 2019-11-01

**Authors:** Olivier Aromatario, Aurélie Van Hoye, Anne Vuillemin, Aude-Marie Foucaut, Jeanine Pommier, Linda Cambon

**Affiliations:** 1 UMR 6051 ARENES, EHESP, 20 avenue George Sand, La Plaine Saint Denis, 93210 Paris, France; 2 EA 4360, APEMAC, Université de Lorraine, Nancy, France; 3 Université Côte d’Azur, LAMHESS, Nice, France; 4 Université Paris 13, Sorbonne Paris Cité, Laboratoire Educations et Pratiques de Santé (LEPS) EA 3412, UFR SMBH, F-93017, Bobigny, France; 5 Chaire de Prévention, ISPED, Centre de recherche U1219 Bordeaux Population Health, Université de Bordeaux, Bordeaux, France

**Keywords:** E-health, Apps, Framework, Theory of change, Healthy eating, Physical activity, Complex interventions, Prevention, Behavior, Effectiveness

## Abstract

**Background:**

Connected health devices and applications (referred to hereafter as “SDApps” - Smart devices and applications) are being portrayed as a new way for prevention, with the promise of accessibility, effectiveness and personalization. Many effectiveness evaluations (experimental designs) with strong internal validity exist. While effectiveness does appear to vary, the mechanisms used by these devices have not yet been thoroughly investigated. This article seeks to unpack this black box, and describes the process of elaboration of an intervention theory for healthy eating and physical activity SDApps. It includes a set of requirements relative to their impact on social health inequalities.

**Methods:**

To build this theory, we drew on theory-driven approaches and in particular on the theory of change (ToC) method. To this end, we developed a cumulative and iterative process combining scientific data from the literature with knowledge from experts (researchers and practitioners) and from patients or users. It was a 3-step process, as follows: 1 - identifying the evidence base; 2 - developing the theory through design intervention and creating realistic expectations, including in our case specific work on social health inequalities (SHIs); 3 - modeling process and outcome.

**Results:**

We produced an evidence-based theory according to the ToC model, based on scientific evidence and knowledge from experts and users. It sets out a causal pathway leveraging 11 key mechanisms - theoretical domains - with which 50 behavior change techniques can be used towards 3 ultimate goals: Capacity, Opportunity, Motivation – Behavior (COM-B). Furthermore, the theory specifically integrates requirements relative to the impact on SHIs.

**Conclusions:**

This theory is an aid to SDAapp design and evaluation and it can be used to consider the question of the possible impact of SDApps on the increase in inequalities. Firstly, it enables developers to adopt a more overarching and thorough approach to supporting behavior change, and secondly it encourages comprehensive and contributive evaluations of existing SDApps. Lastly, it allows health inequalities to be fully considered.

## Background

Connected health devices and applications (SDApps) are being portrayed as a new way for prevention, with the promise of both effectiveness and personalization to support healthy behaviors, in particular physical activity, healthy eating and wellbeing of any kind (goal-setting apps, self-monitoring, cues for healthy eating, etc.) [1]. However, while many effectiveness evaluations with strong internal validity exist (experimental designs), they have not reached a consensus [2–4]. Effectiveness appears to vary [2–4]. The mechanisms by which these SDApps actually change or do not change a person’s behavior have yet to be fully explained in relation to behavior change theories, and their impact on social health inequalities, which is a major public health issue, has been little investigated [2–4]. There is however a general agreement that these “patient-focused” SDApps [5] are more than mere tools, and that they are indeed complex interventions [4] which contain interacting components. As such, they should be evaluated accordingly [3, 4]. International guidelines based on the Health Technology Assessment (HTA) [6] do acknowledge that these devices have a combination of individual, social and environmental factors [7], revealing their complex dimension, but there is no precise guidance on how to understand how such devices may influence behavior.

In view of this, the guidance from the Medical Research Council (MRC) [8] on the evaluation of complex interventions may supplement and enrich HTA approaches by offering a method for analyzing SDAapp effectiveness in behavior change. This guidance stresses that every intervention comprises components which interact with each other and with the context in which the intervention is delivered. This encourages researchers to evaluate intervention processes in addition to effectiveness, by revealing the causal chains involving the intervention components which lead to outcomes. This is what is called black box evaluation [9].

As regards effectiveness, “evaluators need to understand not just whether, but how and why an intervention has a particular effect, and which parts of a complex intervention have the greatest impact on outcomes” [10]. The MRC guidance highlights the benefit of theory-driven approaches for evaluating and designing interventions [10]. These approaches [11–14] aim to examine how hypothesized causal chains play out in practice and how a program brings about specific long-term outcomes through a logical sequence of intermediate outcomes, thus explaining a causal pathway [10]. Should these causal hypotheses be confirmed, there would be grounds for causal inference as evidence of contribution. In this respect, theory-driven approaches can increase our understanding of how an intervention contributes to an outcome rather than demonstrating an attributive causality through counterfactual comparison [11, 12, 15]. This contributive analysis, which is included in theory-driven approaches, can strengthen randomized controlled trials (RCTs) by building and validating program theories of interventions that are then empirically tested [16].

One of the theory-driven approaches is the theory of change (ToC). This involves studying the parts of an intervention separately from the elements of the context in which the intervention is delivered [10, 17, 18]. It involves outlining the key ingredients or components [19, 20] and distinguishing them from the context, so that their real contribution to outcomes can be examined. To build this ToC, many generalist explanatory classical and interpretative theories could be selected and used according to the theme, the beneficiaries of the intervention, the nature of the intervention or the settings: psychosocial and organizational theories, process models, determinant frameworks, etc. They are selected and combined in order to contribute to designing the ToC which describes how the intervention is supposed to work. A logic diagram, based on the science and on stakeholder expertise, here referred to as intervention theory, is thus set out, displaying the key active components of the intervention, the mechansisms activated by them, the intermediate and final outcomes and the contextual factors which may have an effect. This logic diagram, which embodies the theory, hypothesizes the inferences between these components (based on classical explanatory theories, models, frameworks), and between these components and the expected outcomes of the intervention. These hypotheses are validated according to the extent to which the theory - highlighting the causal pathway - corresponds to what is observed in practice [10, 12, 21].

As health SDApps which have been designed for behavior change can be considered as interventions, the ToC approach is appropriate for exploring the conditions for their effectiveness.

By basing the design and evaluation of SDApps on an intervention theory, we can immediately bring to light the mechanistic hypotheses underlying the manufacture of the devices. In this way, we can better assess these devices and thus gauge how effective they are [8] thanks to the knowledge produced about each component’s contributivity to the outcome. This is where the concepts of key functions developed by Hawe [22] and research on transferability come into play [23].

The aim of the OCAPREV project (Objets Connectés et Application en PREVention - Connected devices and applications for prevention) is to elaborate an intervention theory for SDApps that support healthy eating and physical activity for adults over 18, with the emphasis on social health inequalities (SHI). This article sets out how the theory was elaborated using the ToC model, and presents the theory as a potential framework for designing and evaluating behavior change SDApps in the two above-mentioned areas. The theory takes into account the important issue of social health inequalities. This is in line with research by Latulippe, who [24] has shown that the digital divide in eHealth is a serious barrier and contributes greatly to social health inequalities. Ethnicity and low income are the most commonly used characteristics to identify people at risk of SHI. The most promising actions for reducing SHI via eHealth are those aiming for universal access to eHealth tools, those taking account of users’ literacy levels, those creating eHealth tools that respect the cultural attributes of future users, and those encouraging the participation of people at risk of SHI. This should therefore be borne in mind when designing SDApps and defining their purpose, but also when assessing them in terms of their use, acceptability and effectiveness. The theory therefore specifically covers these dimensions.

## Methods

In the ToC model [10], a causal pathway is an articulated series of components: expected changes (e.g. 30 min of daily physical activity per day); individual or socio-ecological preconditions for achieving the intended results (e.g. sufficient physical ability, knowledge of what physical activity means etc.); activities to complete in order to ensure the preconditions are met (e.g. the types of intervention or necessary changes to the setting); and the resources and set-up required for implementing the activities (e.g. intellectual, cognitive, human, financial, organizational resources). The preconditions are intermediate milestones between the activities and the expected outcomes, which are called mechanisms by some authors [25].

The above can be represented in a ToC map or narrative for testing. The theory-building process [26] is one that is cumulative and iterative, combining scientific data from the literature (empirical and theoretical) with knowledge from experts (researchers and practitioners) and from patients or users [27].

We followed Da Silva’s 3-step process (5) which supplemented and adapted the steps set out in the MRC guidance [28] for the ToC: Step 1 - identifying the evidence base; Step 2 - developing the theory through design intervention and creating realistic expectations, including in our case specific emphasis on SHIs; Step 3 - modeling process and outcome. It aims to guide the iterative development of a theory of change: combining outcomes, mechanisms and components.

**Fig. 1:**
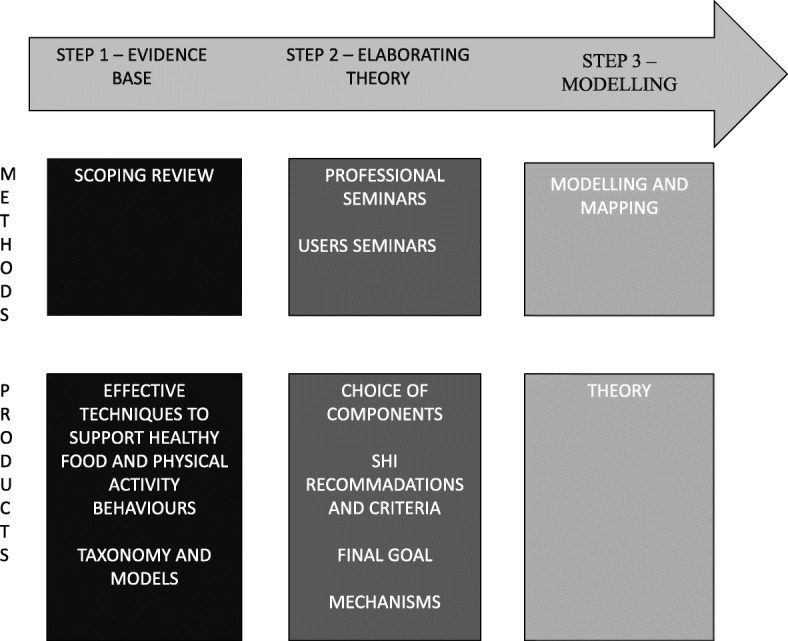
Flow chart of intervention mapping

The rest of this section deals with the methods used in each of the three steps, and how they have helped advance the elaboration of the theory, as shown in Fig. 1.

### Step 1 - identifying the evidence base

This step aims to identify and select data from the literature that could help stakeholders involved in phase 2 in designing the intervention theory. These data may be related to SDApps evaluations or theoretical or methodological frameworks, etc. To select these data, we conducted a scoping review which explored how the SDApps were evaluated [3] and whether the SDApps were based on theory [3]. According to our findings, experimental modes were mostly used without any evaluation of the process involved nor any theory-driven approach for unpacking the black box of conditions for the effectiveness of such apps. We observed that few SDApps are based on theory [3]. The few theory-based SDApps which were identified drew on classical interpretative theories from psychosociology, and in particular on certain behavior-change theories, without any details on the way the choice of theories was made [29]. In particular, we analyzed the behavior change techniques (BCTs) used, considered as components of the SDApps. The BCTs used by the SDApps were underreported, so it is difficult to compare the apps with each other in order to make recommandations. This is why we added the literature describing intervention methods in the fields of physical activity and diet, even if they are not focused on e-prevention. We chose three of them because the interventions were described with BCTs or because they sum up the main effective characteristics in the areas of diet and physical activity:

- An analysis of two systematic reviews of interventions to increase physical activity and healthy eating using a taxonomy of behavior-change techniques: the CALO-RE taxonomy [30].
- A systematic review [31] related to the effectiveness of eHealth interventions. It highlights activities identified as being effective in a number of trials for e-health interventions.
- A meta-regression, undertaken by Michie [32], highlighting 5 effective self-regulation techniques in healthy eating and physical activity interventions.

Not all of this research covered e-interventions, but some of it was selected to help us examine which components to include in the intervention theory. Moreover, certain results highlight specific models for reporting techniques to help change people’s behavior: the Behavior-Change Techniques (BCTs) Taxonomy [33]. Indeed, there are over 80 theoretical models which could be used, alone or in combination [29], to develop behavioral interventions [34] and covering more or less different dimensions. It is a complex task to choose one over the other. Building an intervention theory involves combining other classical theories, frameworks and models to define the most accurate intervention theory. Therefore, by opting for a universal taxonomy, we can strengthen the theory-based approach to designing an intervention as it allows us to avoid having to choose one model over another. Michie’s taxonomy was chosen because it defined the BCTs with interdisciplinary and international behavior change experts. The BCTs are the active ingredients within the intervention designed to change behavior. “They are discrete, low-level components of an intervention that on their own have potential to change behavior, and are observable and replicable” [35] (e.g.: BCT 1.1: Goal setting (behavior), Set or agree on a goal defined in terms of the behavior to be achieved). These BCTS are universal, so they could be used across behavioral interventions. According to Michie et al, these BCTs trigger mechanisms which are defined as “processes through which a BCT affects behavior”, and which influence behavior, defined as “anything a person does in response to internal or external events” [36]. So this step allows us to rigorously define the different components of our theory and the most effective BCTs on healthy eating and physical activity [30].

### Step 2 - developing the theory

In keeping with theory-driven approaches, we put together 4 expert focus groups and one user focus group in order to elaborate the intervention theory. An e-Delphi method [37] was then used to enable all the expert panellists to reach a consensus by validating the findings of the focus groups.

#### Composition of the panel

We formed two groups: a multidisciplinary professional group and a user group with potential users of SDApps for healthy eating and physical activity purposes. The former was composed of 20 individuals: researchers with various backgrounds including learning sciences, sports sciences, public health, psychology and sociology; healthcare and prevention practitioners; professionals from fields relating to healthy eating and physical activity, with a general practitioner, a private dietician, and two health educators including a psychologist; a user representative; and a smartphone app designer (private firm).

The user group was composed of 12 individuals, 5 men and 7 women above the age of 18, who were interested and who volunteered. They were included because they were part of a cardiovascular disease prevention network [38].

**Fig. 2:**
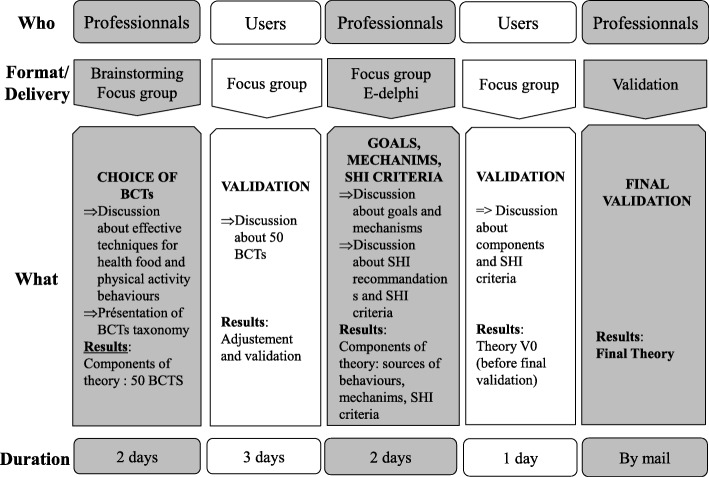
Process of elaborating the theory

The groups worked independently of each other and were run (brainstorming, focus groups) to help build a theory using the theoretical and technical models deemed effective in step 1. This process in step 2 is shown in Fig. 2. For each sequence, it shows the panel involved, the format used, the objectives and the time allocated (except when the work was carried out by email).

#### The choice of BCTs

The BCTs were selected during a 2-day professional seminar and during the first user seminar. This selection was based on two main criteria defined by the group of experts. The first selection criterion was selected on ethical principles. To define them, we based the work of the group on ethical health promotion criteria from Carter [39]. Two main principles conditioning the others are the non-coercion and the non-stigmatization principles because coercion and stigmatization are not compatible with the aim of health promotion: empowerment. For example, the “Punishment” BCT was excluded. The second selection criterion was selected on the suitability of the BCTs for a simple application: the group had to assess each BCT through one question: is it possible to keep this BCT in a simple application? When the answer was no, the BCT was excluded. For example, “Cue signalling reward” (Identify an environmental stimulus that reliably predicts that reward will follow the behavior (includes ‘Discriminative cue’)) was excluded because it requires a structural modification of the environment.

Consensus was systematically sought at all times within both groups, and then the opinions of both groups were debated until a consensus was reached. The consensus results were consistent with the results of the three publications cited in step 1.

#### The choice of final goals

We decided not to define the final goals based on behavioral recommendations (e.g. eat 5 fruit and veg a day), for two reasons. The first is to apply the principle of proportionate universalism [40], which reduces SHIs. This principle states that actions must be universal, but with a scale and intensity that is proportionate to the level of disadvantage. It is consistent with the principle of avoiding normativity in health promotion [41]. The second is the known incapacity of SDApps to provide sustainable behavioral changes without complementary environmental interventions to support the changes. This is the weakness of behavioral intervention. Nevertheless, we think that proper SDApps could contribute to supporting changes in individual behaviour resources such as motivation, self-efficacy and skills, according to users’ needs. So we decided to consider these prerequisites to behavior changes as final goals. This led us to use the “behavior system”, which forms the hub of the behavior change wheel [42] (COM-B model). This generalist framework associates behavior change, whatever form it may take, with three sources of behavior: capability, opportunity, and motivation. These sources of behavior “interact to generate behavior that in turn influences the components” [42]. On the basis of this model, the research team linked each BCT to a source of behavior [42]. To do this, 2 team members (LC, OA) each carried out the operation independently of one another, and then compared their choices during two work sessions. Each choice was discussed until a consensus was reached. The work was validated by the focus groups during the second 2-day seminar.

#### The choice of intermediate outcomes, called mechanisms

In the same way as for the BCT taxonomy, we used the twelve theoretical domains described by Michie [43] in order to identify the mechanisms activated by the BCTs. Indeed, each theoretical domain, from classical psychosocial theories [43], could be considered as a mechansim according to Michie’s definition of mechanisms [33]: “the processes by which a behavior change technique regulates behavior”. They can be considered as intermediate outcomes [14] between the BCTs and the behavior.

To determine the connections between these mechanisms, the BCTs and the sources of behavior, the work involved four main tasks: i) Firstly, the multidisciplinary study team shared a common understanding of the 12 theoretical domains [43]; ii) Secondly, using an e-Delphi method [37] in 3 rounds of questions (8 respondents including 5 researchers), we asked the professional group to allocate one or several mechanisms (i.e. a theoretical domain) to each BCT. The procedure was based on an online self-questionnaire allowing these linkages. On this basis we selected the linkage mechanism(s) - BCTs chosen by at least half of the experts - and proceeded to a second round asking the group to validate or adjust them. We again selected the linkage mechanism(s) - BCTs chosen by half of the respondents; iii) Thirdly, during the 2nd user seminar, we asked the user group to validate or adjust the linkage mechanism(s) - BCTs produced by professionals. They validated all of them and added several comments which we then added into the e-Delphi software program [37]; iv) Finally, in the 3rd round of the e-Delphi procedure, we asked the professional group to validate the list of linkage mechanism(s) – BCTs adjusted. They did so.

#### Integrating the question of social health inequality (SHI)

To avoid the possibility of increasing SHIs with SDApps, the professional and user groups analyzed each BCT according to an analytical grid for interventions aimed at reducing or not increasing SHIs, designed by Guichard and Ridde [44]. This grid describes 51 criteria across 5 categories applicable to face-to-face or collective health promotion interventions: action planning, action implementation, evaluation, sustainability and empowerment. Drawing on this work, two members of the research team (LC, OA) drew up an initial list of SHIs requirements, recommendations for each BCT, and areas of vigilance to be covered by the theory.

This list was then presented to the professionals for discussion. It included research relating to the “access/literacy/culturality” trio used to address SHIs in the field of e-health [24, 45]. This discussion was conducted using a focus-group method during the second 2-day professional seminar. The work was then validated by the user group during the second user seminar (see Fig. 2).

### Step 3 - Modelling process and outcome

The purpose of this step is to represent, in an understandable way, the interaction between the intervention components, mechanisms and outcomes. An example of a ToCmap on peer counselling for maternal depression intervention has been presented by De Silva [10]. All the intervention theory elements were then modeled by the Xmind® software to present them in the form of a map.

## Results

The results of this multidisciplinary work based on the ToC model enabled the elaboration of an intervention theory which articulates:

- BCTs to interpret the SDApp activities;
- each BCT which generates mechanisms or preconditions for the expect outcome as described against Michie’s 11 theoretical domains;
- each mechanism or precondition which influences, alone or in combination, sources of behavior and the final goals of the BCTs;
- areas of vigilance relating to SHIs.

### The intervention theory

**Fig. 3:**
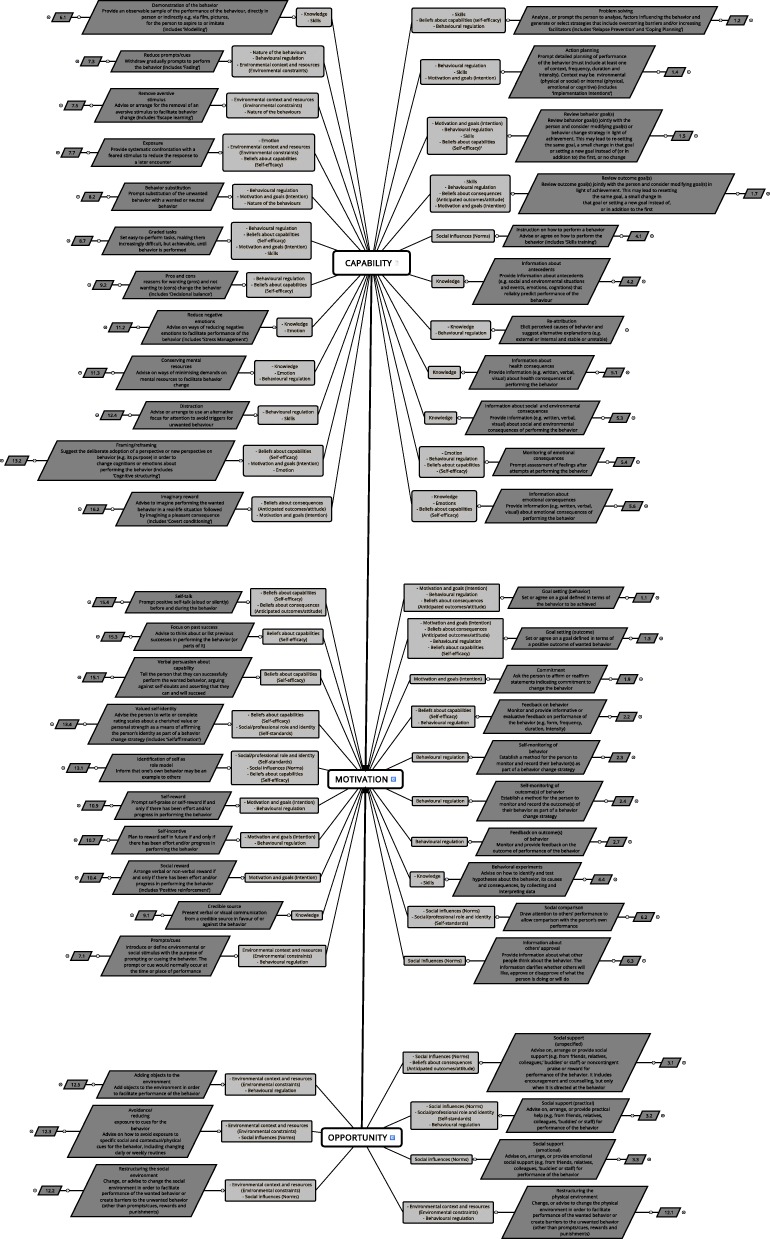
Intervention theory map

Additional file 1: Table S1 shows the whole intervention theory. Figure 3 shows the intervention theory map with the components and inferences linked to the 3 sources of behavior.

#### Behavior change techniques mobilized

Our theory articulates 50 different BCTs. Of the 93 BCTs in the general BCT taxonomy [33], 43 were excluded by the expert panelists (Table 1):

- 11 BCTs were excluded because they are not suitable for application (see step 2 above) or they did not fit with the field of diet and physical activity;
- 32 BCTs were excluded because they did not comply with the two ethical principles.

**Table 1:** BCTs excluded

BCTs excluded	BCTs (corresponding numbers in Michie’s Taxonomy)
BCTs excluded because they are not suitable with application specificities or did not fit with the field (diet and physical activity).	1.6 Discrepancy between current behavior and goal
	2.6 Biofeedback
	8.1 Behavioral practice/rehearsal, 8.3 Habit formation
	10.1 Material incentive (behavior), 10.2 Material reward (behavior), 10.3 Non-specific reward
	11.1 Pharmacological support
	13.3 Incompatible beliefs, 13.5 Identity associated with changed behavior
	15.2 Mental rehearsal of successful performance
BCTs excluded because they did not comply with the two ethical principles	1.8 Behavioral contract
	2.1 Monitoring of behavior by others without feedback, 2.5 Monitoring of outcome(s) of behavior without feedback
	5.2 Salience of consequences, 5.5 Anticipated regret
	7.2 Cue signalling reward, 7.4 Remove access to the reward, 7.6 Satiation, 7.8 Associative learning
	8.4 Habit reversal, 8.5 Overcorrection, 8.6 Generalisation of target behavior
	9.3 Comparative imagining of future outcomes
	10.5 Social incentive, 10.6 Non-specific incentive, 10.8 lncentive (outcome), 10.10 Reward (outcome), 10.11 Future punishment
	11.4 Paradoxical instructions
	12.6 Body changes
	14.1 Behavior cost, 14.2 Punishment, 14.3 Remove reward, 14.4 Reward approximation, 14.5 Rewarding completion, 14.6 Situation-specific reward, 14.7 Reward incompatible behavior, 14.8 Reward alternative behavior, 14.9 Reduce reward frequency, 14.10 Remove punishment
	16.1 Imaginary punishment, 16.3 Vicarious consequences

For 18 out of the 50 BCTs, special recommendations were made about their feasibility and SHIs (See details in Table 2).

**Table 2:** BCTs included with special recommendations

BCTs with special recommendations made about their feasibility and SHIs	1.1 Goal setting (behavior), 1.3 Goal setting (outcome), 1.5 Review behavior goal(s), 1.7 Review outcome goal(s), 1.9 Commitment
	2.2 Feedback on behaviour, 2.3 Self-monitoring of behaviour, 2.4 Self-monitoring of outcome(s) of behaviour
	3.1 Social support (unspecified), 3.2 Social support (practical)
	4.1 Instruction on how to perform the behavior
	6.2 Social comparison, 6.3 Information about others’ approval
	7.1 Prompts/cues, 7.3 Reduce prompts/cues, 7.5 Remove aversive stimulus, 7.7 Exposure
	8.2 Behavior substitution

Of the theory’s 50 BCTs, 42 (84%) corresponded to the CALO-RE taxonomy. The remaining BCTs (16%) did not, due to the fact that the CALO-RE taxonomy predated the BCT taxonomy and did not always use the same terminology, which hampered the matching up process. In addition, the theory integrates the 5 techniques which have been identified as being more effective than others by Michie’s meta-regression [32].

#### Theoretical domains as mechanisms

As defined by the overall consensus of the e-Delphi process, the 50 BCTs selected influence 11 theoretical domains which work as the mechanisms or preconditions relating to the sources of behavior (Table 3): 24 BCTs influence Behavioral regulation, 15 BCTs influence Beliefs about capabilities (Self-efficacy), 13 BCTs influence Motivation and goals (Intention), 10 BCTs influence Knowledge, 9 influence Social influences (Norms), 8 BCTs influence Environmental context and resources (Environmental constraints) and 8 BCTs also influence Skills, 6 BCTs influence Beliefs about consequences (Anticipated outcomes/attitude) and 6 BCTs also influence Emotion, 4 BCTs influence the Social/professional role and identity (Self-standards) and 3 BCTs influence the Nature of the behaviors. No BCT influences the Memory, attention and decision processes. Overall, 40% of the BCTs influence 2 theoretical domains, 28% influence a single theoretical domain, 24% influence 3, and 8% of the BCTs influence 4 together.

**Table 3:** Theoretical domains influenced by BCTs

Theoretical domains	BCTs (corresponding numbers in Michie’s Taxonomy)
Behavioral regulation	1.1/1.2/1.3/1.4/1.5/1.7/2.2/2.3/2.4/2.7/3.2/4.3/5.4/7.1/7.3/8.2/8.7/9.2/10.7/10.9/11.3/12.1/12.4/12.5
Beliefs about capabilities (Self-efficacy)	1.2/1.3/1.5/2.2/5.4/5.6/7.7/8.7/9.2/13.1/13.2/13.4/15.1/15.3/15.4
Motivation and goals (Intention)	1.1/1.3/1.4/1.5/1.7/1.9/8.2/8.7/10.4/10.7/10.9/13.2/16.2
Knowledge	4.2/4.3/4.4/5.1/5.3/5.6/6.1/9.1/11.2/11.3
Social influences (Norms)	3.1/3.2/3.3/4.1/6.2/6.3/12.2/12.3/13.1
Environmental context and resources (Environmental constraints)	7.1/7.3/7.5/7.7/12.1/12.2/12.3/12.5
Skills	1.2/1.4/1.5/1.7/4.4/6.1/8.7/12.4
Beliefs about consequences (Anticipated outcomes/attitude)	1.1/1.3/1.7/3.1/15.4/16.2
Emotion	5.4/5.6/7.7/11.2/11.3/13.2
Social/professional role and identity (Self-standards)	3.2/6.2/13.1/13.4
Nature of the behaviors	7.3/7.5/8.2
Memory, attention and decision processes	/

#### Sources of behavior as final goals

Of the 50 BCTs, 23 directly affect capability, 20 directly affect motivation and 7 directly affect opportunity. All the BCTs affecting opportunity and capability also affect motivation [42].

**Fig. 4:**
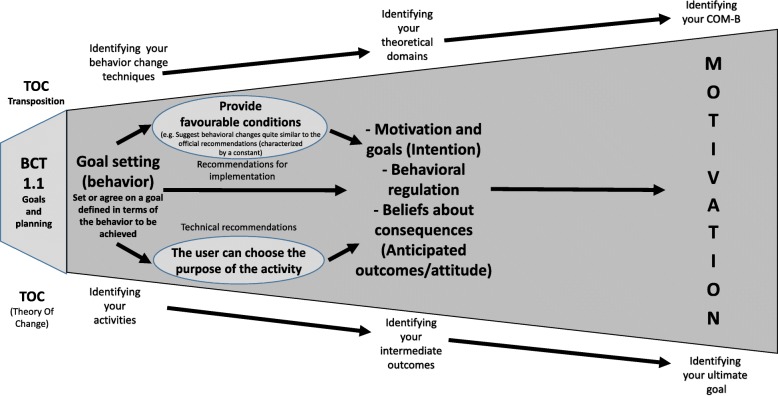
Example of a causal pathway for BCT 1.1

This theory thus highlights mini linear causal pathways, as illustrated in Fig. 4, which are themselves combined. Each BCT’s final goal is articulated with its intermediate outcomes (mechanisms activated). For each BCT, special technical or implementation recommendations (see Additional file 1: Table S1) and ethical or SHI-related requirements were specified (see Additional file 2: Table S2).

### Integration of SHI into the theory

A checklist was established to ensure the integration of the requirements related to not increasing SHIs. It includes 27 criteria taken from Guichard’s and Ridde’s analytical grid [44] adapted to the specificities of SDApps. And 5 further criteria were extracted from Latulippe’s [24] and Berland’s works [45].

Some of the SHI criteria are directly integrated as BCTs (e.g. the criterion “the application features activities relating to social support” corresponds to 3.1, 3.2, 3.3 Social support (unspecified, practical, emotional)). For 17 BCTs we were thus able to set out recommendations focusing on the need to adapt the apps to each user’s needs, differences and health literacy. This includes the possibility for the user to choose not to perform the activity (corresponding numbers in Michie’s Taxonomy: 1.9/2.2/2.3/2.4/3.1/3.2/4.1/6.2/6.3/7.1/7.5/7.7/8.2), to choose how often the activity is suggested (corresponding numbers in Michie’s Taxonomy: 1.5/1.7/7.3) and to choose the purpose of the activity (corresponding numbers in Michie’s Taxonomy: 1.3). Lastly, other criteria spanned the intervention theory (e.g. the language used is easy to understand by everyone) (see Additional file 2: Table S2).

We thus produced a specific grid containing 32 additional criteria divided into 10 categories relating to physical, geographical and financial issues and also to user literacy. It details whether criteria are related to a particular BCT and to some elements in the app store and/or in the app itself (e.g. the app was designed with help from a user group, or the app’s advice and activities take users’ financial constraints into account).

## Discussion

This article sets out how an intervention theory has been elaborated to analyze/evaluate or build a SDApp that supports behavior change relating to healthy eating and physical activity. To achieve this, we performed a participative process, based on the ToC model. It combined some scientific evidence on effective supporting interventions and techniques, some theoretical frameworks from health psychology, and expertise from practitioners, researchers, and SDApp users. We produced an evidence-based theory revealing a causal pathway leveraging 11 key mechanisms - theoretical domains - with which 50 behavior change techniques (BCTs) can be used toward 3 final goals (COM-B). Furthermore, the theory specifically considered 32 criteria, in order not to increase SHIs. Lastly, 48% of the techniques used in the theory correspond to the effective CALO-RE taxonomy and the theory integrates the 5 most effective techniques identified in the literature on healthy eating and physical activity, which enhances its quality.

The theory developed could be useful in various ways. By using the BCTs, it allows SDApps to be designed and tailored to the type of behavioral goals sought: increasing motivation, developing capabilities, increasing the opportunities for behavior change. In this respect, the theory offers an SDApp design framework which is not based on behavioral standards as such, but rather on a real objective to provide tailored support according to users’ needs. Indeed, most applications use effective techniques to increase motivation (self-reporting, goal defining, reward providing, etc.) [4, 46, 47]. They do not address, or barely address, the techniques which influence capabilities and the opportunity to change. This could be why these applications produce short-term results. It could also be why they are swiftly abandoned (mostly after having been used 10 times): they are used by already motivated users [48–50]. This theory can therefore make a real difference in the provision of SDApps.

In addition, the intervention theory provides a comprehensive and contributive evaluation of existing behavior change SDApps. Each causal pathway is a hypothesis for supporting users and can be assessed. The theory can be used to unpack each application’s black box and test how it corresponds to the causal pathways (BCT-mechanism-final goal) described. This allows further in-depth analysis of the conditions for their effectiveness: which BCTs should be used for setting higher final goals? Which mechanisms (self-regulation, self-efficacy, the knowledge gained, etc.) should be used by which type(s) of BCT? What results are to be expected with such BCTs? Lastly, as indicated earlier, special emphasis has been placed on SHIs, with 32 criteria to take into account in order to be sure not to increase them. This theory can therefore offer an evaluation framework to complement the experimental designs traditionally used to evaluate SDApps promoting active lifestyles and healthy eating, by asking the following question: in what conditions do SDApp components contribute to outcomes? In this respect, the theory complements the HTA framework on the theoretical effectiveness and reliability of the data used by the apps and contributes to the reflection on process evaluation by the MRC guideline [8] . To sum up, this theory could allow app developers to improve the design of apps or parts of apps. Moreover, it could allow evaluators or practitioners (willing to select the most accurate applications to their patients for example) to assess the black box of existing apps: components and relationship between them, mechanisms and goals.

### Strengths and limitations of the process

In terms of methods, quality is ensured during the process by combining scientific knowledge with strong internal validity, proven theoretical frameworks, and expert and user consensus. The professionals drew on scientific evidence to build the theory. They also benefited from users’ feedback. They examined each hypothesis in a real-life setting and in terms of desirability and sustainability. The most visible result of this knowledge matrix is borne out in the recommendations and conditions of use, thus allowing the highly complex dimension of the subject to be properly addressed. Instead of focusing on behavioral standards, which are controversial in health promotion and in particular SHIs production, the theory allows a constant adaptation to individual and contextual factors.

There are however some limitations in our work. Although the theory involves a thorough process (ToC model, combined expertise, use of scientific evidence), and uses certain techniques corresponding to those identified in the literature, and despite it bringing out the solidity of the inferential hypotheses, it has yet to be validated. It would therefore be interesting to validate causal pathways included in the theory by using and evaluating either existing SDApps involving the theory’s BCTs, or specific SDApps built on the theory. The intervention theory’s solidity could thus be confirmed.

Another limit is relative to the choice of sources of behaviors as ultimate goals. In the theory, the intercorrelation between physical activity and healthy eating habits and how the BCTS involved influence each other are not explained. Nonetheless, these relationships could be addressed in the validation study mentioned above.

Finally, the last limit concerns the parameters not included in the theory. Indeed, to be effective, a SDApp has to be adopted and used. We did not take into account the parameters influencing the acceptability and the usability of SDApps. This is a specific and different research field wich could contribute to improving the intervention theory.

## Conclusions

The aim of this work was to build an evidence-based intervention theory for designing and evaluating SDApps that support behavior change relating to healthy eating and physical activity. A key feature is that it specifically takes into account certain criteria in order to consider SHIs issues. The research draws on a thorough process based on theory-driven approaches and the contributive analysis paradigm. The theory elaborated offers a solid reflexivity framework for evaluating existing applications and for designing new ones. As such, it offers new and more comprehension methods for evaluating prevention SDApps, and these methods address their complexity as promoted by MRC guidance on process evaluation. Developers of SDApps must therefore work with the different categories of health prevention experts, including in behavioural sciences.

## Supplementary information

**Additional file 1: Table S1.** Intervention Theory.

**Additional file 2: Table S2.** SHI criteria for SDApps.

## Data Availability

Not applicable.

## Abbreviations

Apps: Applications
BCT: Behavior Change Technique
COM-B: Capability, Opportunity, Motivation-Behavior
HTA: Health Technology Assessement
MRC: Medical Research Council
SDApps: Health smart devices and applications
SHI: Social Health Inequalities
ToC: Theory of Change
