# Supplementary material for: Using theory of change to develop an intervention theory for designing and evaluating behavior change SDApps for healthy eating and physical exercise: the OCAPREV theory
Source: BMC Public Health. 2019 Nov 1;19:1435. doi: 10.1186/s12889-019-7828-4 (PMC6824130; doi:10.1186/s12889-019-7828-4)
Supplement: Supplementary file 1 — Additional file 1: Table S1. Intervention Theory. [file 12889_2019_7828_MOESM1_ESM.pdf]

Table S1: Intervention theory

	BCT	RECOMMANDATIONS FOR IMPLEMENTATION	BCT	RECOMMANDATIONS FOR IMPLEMENTATION	BCT	RECOMMANDATIONS FOR IMPLEMENTATION	BCT	RECOMMANDATIONS FOR IMPLEMENTATION
	1.2		4.1		4.2		6.1	
**BEHAVIOUR CHANGE TECHNIQUES (Activities)**	**Problem solving** Analyse , or prompt the person to analyse, factors influencing the behavior and generate or select strategies that include overcoming barriers and/or increasing facilitators (includes 'Relapse Prevention' and 'Coping Planning')	Suggest or to enable to higlight influencing factors of behaviors: physical environment, social environment, own skills. Suggest strategies / tips according to these factors.	**Instruction on how to perform a behavior** Advise or agree on how to perform the behavior (includes 'Skills training')	Suggest concrete tips and tricks in different contexts Important : Adapt triks and tips according to individual characteristics investigated by Apps	**Information about antecedents** Provide information about antecedents (e.g. social and environmental situations and events, emotions, cognitions) that reliably predict performance of the behaviour	Highlight positive elements (e.g. behavioral intentions, perceived competence, identification of benefits, emotions) rather than negative ones. Provide them not at the beginning of the process. Beware of stigmatization and guilt process.	**Demonstration of the behavior** Provide an observable sample of the performance of the behaviour, directly in person or indirectly e.g. via film, pictures, for the person to aspire to or imitate (includes 'Modelling')	Provide videos and tutorials
**Technical recommendations**			The user can choose to use or not the option					
**THEORITICAL DOMAINS (Intermediate outcomes)**	(2) Skills		(9) Social influences (Norms)		(1) Knowledge		(2) Skills	
	(4) Beliefs about capabilities (Self-efficacy)						(1) Knowledge	
	(11) Behavioural regulation							
**COM-B (ultimate outcomes)**	CAPABILITY		CAPABILITY		CAPABILITY		CAPABILITY	

	Table S1: Intervention theory											
	BCT	RECOMMANDATIONS FOR IMPLEMENTATION	BCT	RECOMMANDATIONS FOR IMPLEMENTATION	BCT	RECOMMANDATIONS FOR IMPLEMENTATION	BCT	RECOMMANDATIONS FOR IMPLEMENTATION	BCT	RECOMMANDATIONS FOR IMPLEMENTATION	BCT	RECOMMANDATIONS FOR IMPLEMENTATION
**BEHAVIOUR CHANGE TECHNIQUES (Activities)**	1.4		1.5		1.7		4.3		5.1		5.3	
	**Action planning** Prompt detailed planning of performance of the behavior (must include at least one of context, frequency, duration and intensity). Context may be nvironmental (physical or social) or internal (physical, emotional or cognitive) (includes 'Implementation Intentions')	To propose gradual stages and deadline	**Review behavior goal(s)** Review behavior goal(s) jointly with the person and consider modifying goal(s) or behavior change strategy in light of achievement. This may lead to re-setting the same goal, a small change in that goal or setting a new goal instead of (or in addition to) the first, or no change	Establish a recurrence according to the will of the user, just suggest and not impose it (push questions form)	**Review outcome goal(s)** Review outcome goal(s) jointly with the person and consider modifying goal(s) in light of achievement. This may lead to resetting the same goal, a small change in that goal or setting a new goal instead of, or in addition to the first	Establish a recurrence according to the will of the user, just suggest and not impose it (push questions form)	**Re-attribution** Elicit perceived causes of behavior and suggest alternative explanations (e.g. external or internal and stable or unstable)	Cause the user to think about causes and resaons of behaviors by suggestions (e.g. external or internal, ...)	**Information about health consequences** Provide information (e.g. written, verbal, visual) about health consequences of performing the behavior	Use a positive approach, not stigmatizing, and requiring a thoughtful decision	**Information about social and environmental consequences** Provide information (e.g. written, verbal, visual) about social and environmental consequences of performing the behavior	In order to maintain autonomy, remind users the importance to be able to adapt themselves to their physical and social environement and act on it in order to change it in a favorable way
**Technical recommendations**			The user chooses the recurrence		The user chooses the recurrence							
**THEORITICAL DOMAINS (Intermediate outcomes)**	(11) Behavioural regulation		(11) Behavioural regulation		(2) Skills		(1) Knowledge		(1) Knowledge		(1) Knowledge	
	(2) Skills		(6) Motivation and goals (Intention)		(11) Behavioural regulation		(11) Behavioural regulation					
	(6) Motivation and goals (Intention)		(2) Skills		(5) Beliefs about consequences (Anticipated outcomes/attitude)							
			(4) Beliefs about capabilities (Self-efficacy)		(6) Motivation and goals (Intention)							
**COM-B (ultimate outcomes)**	CAPABILITY		CAPABILITY		CAPABILITY		CAPABILITY		CAPABILITY		CAPABILITY	

Table S1: Intervention theory

	BCT	RECOMMANDATIONS FOR IMPLEMENTATION	BCT	RECOMMANDATIONS FOR IMPLEMENTATION	BCT	RECOMMANDATIONS FOR IMPLEMENTATION	BCT	RECOMMANDATIONS FOR IMPLEMENTATION	BCT	RECOMMANDATIONS FOR IMPLEMENTATION	BCT	RECOMMANDATIONS FOR IMPLEMENTATION
	5.4		5.6		7.3		7.5		7.7		8.2	
**BEHAVIOUR CHANGE TECHNIQUES (Activities)**	**Monitoring of emotional consequences** Prompt assessment of feelings after attempts at performing the behavior	Position him/herself on a playful scale . If possible, enable users to position their feelings after each progress.	**Information about emotional consequences** Provide information (e.g. written, verbal, visual) about emotional consequences of performing the behavior	In a positive and rewarding approach	**Reduce prompts/cues** Withdraw gradually prompts to perform the behavior (includes 'Fading')	With methods based on progressive development of skills and empowerment	**Remove aversive stimulus** Advise or arrange for the removal of an aversive stimulus to facilitate behavior change (includes 'Escape learning')	Allow to delete the functions that could be repulsive with time or with the progression of users	**Exposure** Provide systematic confrontation with a feared stimulus to reduce the response to a later encounter	Enable to try sports, to visit equipments, etc	**Behavior substitution** Prompt substitution of the unwanted behavior with a wanted or neutral behavior	Enable access to appropriate tricks or suggestions according to the different situations
**Technical recommendations**					The user chooses the recurrence		The user can choose to use or not the option		The user can choose to use or not the option		The user can choose to use or not the option	
**THEORITICAL DOMAINS (Intermediate outcomes)**	(10) Emotion		(1) Knowledge		(12) Nature of the behaviours		(8) Environmental context and resources (Environmental constraints)		(10) Emotion		(11) Behavioural regulation	
	(11) Behavioural regulation		(10) Emotion		(11) Behavioural regulation		(12) Nature of the behaviours		(8) Environmental context and resources (Environmental constraints)		(6) Motivation and goals (Intention)	
	(4) Beliefs about capabilities (Self-efficacy)		(4) Beliefs about capabilities (Self-efficacy)		(8) Environmental context and resources (Environmental constraints)				(4) Beliefs about capabilities (Self-efficacy)		(12) Nature of the behaviours	
**COM-B (ultimate outcomes)**	CAPABILITY		CAPABILITY		CAPABILITY		CAPABILITY		CAPABILITY		CAPABILITY	

Table S1: Intervention theory

	BCT	RECOMMANDATIONS FOR IMPLEMENTATION	BCT	RECOMMANDATIONS FOR IMPLEMENTATION	BCT	RECOMMANDATIONS FOR IMPLEMENTATION	BCT	RECOMMANDATIONS FOR IMPLEMENTATION	BCT	RECOMMANDATIONS FOR IMPLEMENTATION	BCT	RECOMMANDATIONS FOR IMPLEMENTATION
	8.7		9.2		11.2		11.3		13.2		16.2	
**BEHAVIOUR CHANGE TECHNIQUES (Activities)**	**Graded tasks** Set easy-to-perform tasks, making them increasingly difficult, but achievable, until behavior is performed	Perform the activity with progressivity and adaptation	**Pros and cons** reasons for wanting (pros) and not wanting to (cons) change the behavior (includes 'Decisional balance')	Provide follow-up and recall (with positive reinforcement)	**Reduce negative emotions** Advise on ways of reducing negative emotions to facilitate performance of the behavior (includes 'Stress Management')	Promote the use of a simple method of stress management (relaxation)	**Conserving mental resources** Advise on ways of minimising demands on mental resources to facilitate behavior change	Enable access to appropriate tricks or suggestions according to the different situations	**Framing/reframing** Suggest the deliberate adoption of a perspective or new perspective on behavior (e.g. its purpose) in order to change cognitions or emotions about performing the behavior (includes 'Cognitive structuring')	Enable to revise the steps of the process and discuss the achievement of objectives (or behavior changes) in order to promote a positive way to consider them and avoid self-blaming	**Imaginary reward** Advise to imagine performing the wanted behavior in a real-life situation followed by imagining a pleasant consequence (includes 'Covert conditioning')	Suggest benefits from wanted behaviors (including by the use of other users' testimonies)
**Technical recommendations**												
**THEORITICAL DOMAINS (Intermediate outcomes)**	(11) Behavioural regulation		(11) Behavioural regulation		(1) Knowledge		(1) Knowledge		(4) Beliefs about capabilities (Self-efficacy)		(5) Beliefs about consequences (Anticipated outcomes/attitude)	
	(4) Beliefs about capabilities (Self-efficacy)		(4) Beliefs about capabilities (Self-efficacy)		(10) Emotion		(10) Emotion		(6) Motivation and goals (Intention)		(6) Motivation and goals (Intention)	
	(6) Motivation and goals (Intention)						(11) Behavioural regulation		(10) Emotion			
	(2) Skills											
**COM-B (ultimate outcomes)**	CAPABILITY		CAPABILITY		CAPABILITY		CAPABILITY		CAPABILITY		CAPABILITY	

	Table S1: Intervention theory											
	BCT	RECOMMANDATIONS FOR IMPLEMENTATION	BCT	RECOMMANDATIONS FOR IMPLEMENTATION	BCT	RECOMMANDATIONS FOR IMPLEMENTATION	BCT	RECOMMANDATIONS FOR IMPLEMENTATION	BCT	RECOMMANDATIONS FOR IMPLEMENTATION	BCT	RECOMMANDATIONS FOR IMPLEMENTATION
	12.4		1.3		1.1		2.2		2.3		2.4	
**BEHAVIOUR CHANGE TECHNIQUES (Activities)**	**Distraction** Advise or arrange to use an alternative focus for attention to avoid triggers for unwanted behaviour	Suggest distractions (game, activities ...)	**Goal setting (outcome)** Set or agree on a goal defined in terms of a positive outcome of wanted behavior	Suggest different goals: medical (BMI) or psychosocial (self-esteem, quality of life). Users choose their own goals.	**Goal setting (behavior)** Set or agree on a goal defined in terms of the behavior to be achieved	Suggest behavioral changes quite similar to the official recommendatio ns (characterized by a constant)	**Feedback on behavior** Monitor and provide informative or evaluative feedback on performance of the behavior (e.g. form, frequency, duration, intensity)	Provide this option but without obligation. The user can consult the feedback when he/her wants.	**Self-monitoring of behavior** Establish a method for the person to monitor and record their behavior(s) as part of a behavior change strategy	If possible, propose a tracker to facilitate the monitoring. Enable the user to choose different options of recording : only data regards to the objectives for example. Provide explanations of the usefulness of this recording.	**Self-monitoring of outcome(s) of behavior** Establish a method for the person to monitor and record the outcome(s) of their behavior as part of a behavior change strategy	If possible, propose a tracker to facilitate the monitoring. Enable the user to choose different options of recording : only data regards to the objectives for example. Provide explanations of the usefulness of this recording.
**Technical recommendations**			The user can choose the purpose of the activity		The user can choose the purpose of the activity		The user can choose to use or not the option		The user can choose to use or not the option		The user can choose to use or not the option	
**THEORITICAL DOMAINS (Intermediate outcomes)**	(11) Behavioural regulation		(6) Motivation and goals (Intention)		(6) Motivation and goals (Intention)		(4) Beliefs about capabilities (Self-efficacy)		(11) Behavioural regulation		(11) Behavioural regulation	
	(2) Skills		(5) Beliefs about consequences (Anticipated outcomes/attitude)		(11) Behavioural regulation		(11) Behavioural regulation					
			(11) Behavioural regulation		(5) Beliefs about consequences (Anticipated outcomes/attitude)							
			(4) Beliefs about capabilities (Self-efficacy)									
**COM-B (ultimate outcomes)**	CAPABILITY		MOTIVATION		MOTIVATION		MOTIVATION		MOTIVATION		MOTIVATION	

	Table S1: Intervention theory											
	BCT	RECOMMANDATIONS FOR IMPLEMENTATION	BCT	RECOMMANDATIONS FOR IMPLEMENTATION	BCT	RECOMMANDATIONS FOR IMPLEMENTATION	BCT	RECOMMANDATIONS FOR IMPLEMENTATION	BCT	RECOMMANDATIONS FOR IMPLEMENTATION	BCT	RECOMMANDATIONS FOR IMPLEMENTATION
**BEHAVIOUR CHANGE TECHNIQUES (Activities)**	2.7		4.4		6.2		6.3		7.1		9.1	
	**Feedback on outcome(s) of behavior** Monitor and provide feedback on the outcome of performance of the behavior	Notify the achievement of the outcome(s), underlining the efforts made. Propose to progress or maintain	**Behavioral experiments** Advise on how to identify and test hypotheses about the behavior, its causes and consequences, by collecting and interpreting data	Suggest simple, rewarding, playful things	**Social comparison** Draw attention to others' performance to allow comparison with the person's own performance	Invite the user to increase awareness of their own progression compared to others	**Information about others' approval** Provide information about what other people think about the behavior. The information clarifies whether others will like, approve or disapprove of what the person is doing or will do	Enable to like others' behaviors. Enable to choose who can see her/his behavioral data and like them	**Prompts/cues** Introduce or define environmental or social stimulus with the purpose of prompting or cueing the behavior. The prompt or cue would normally occur at the time or place of performance	Offer music and material to support physical activity	**Credible source** Present verbal or visual communication from a credible source in favour of or against the behavior	Cite regularly sources of information
**Technical recommendations**					The user can choose to use or not the option		The user can choose to use or not the option		The user can choose to use or not the option			
**THEORITICAL DOMAINS (Intermediate outcomes)**	(11) Behavioural regulation		(1) Knowledge		(9) Social influences (Norms)		(9) Social influences (Norms)		(8) Environmental context and resources (Environmental constraints)		(1) Knowledge	
			(2) Skills		(3) Social/professional role and identity (Self-standards)				(11) Behavioural regulation			
**COM-B (ultimate outcomes)**	MOTIVATION		MOTIVATION		MOTIVATION		MOTIVATION		MOTIVATION		MOTIVATION	

	Table S1: Intervention theory											
	BCT	RECOMMANDATIONS FOR IMPLEMENTATION	BCT	RECOMMANDATIONS FOR IMPLEMENTATION	BCT	RECOMMANDATIONS FOR IMPLEMENTATION	BCT	RECOMMANDATIONS FOR IMPLEMENTATION	BCT	RECOMMANDATIONS FOR IMPLEMENTATION	BCT	RECOMMANDATIONS FOR IMPLEMENTATION
	10.4		10.7		10.9		13.1		15.1		15.3	
**BEHAVIOUR CHANGE TECHNIQUES (Activities)**	**Social reward** Arrange verbal or non-verbal reward if and only if there has been effort and/or progress in performing the behavior (includes 'Positive reinforcement')	Provide regularly also supports	**Self-incentive** Plan to reward self in future if and only if there has been effort and/or progress in performing the behavior	Enable to follow user's progression by a way highlighting his/her active role	**Self-reward** Prompt self-praise or self-reward if and only if there has been effort and/or progress in performing the behavior	Enable to be awarness of its own progress	**Identification of self as role model** Inform that one's own behavior may be an example to others	Maintain a positive approach	**Verbal persuasion about capability** Tell the person that they can successfully perform the wanted behavior, arguing against self-doubts and asserting that they can and will succeed	Highlight each progress and each failure too	**Focus on past success** Advise to think about or list previous successes in performing the behavior (or parts of it)	Highlight past successes, progress made by writing, photos, etc
**Technical recommendations**												
**THEORITICAL DOMAINS (Intermediate outcomes)**	(6) Motivation and goals (Intention)		(6) Motivation and goals (Intention)		(6) Motivation and goals (Intention)		(3) Social/professional role and identity (Self-standards)		(4) Beliefs about capabilities (Self-efficacy)		(4) Beliefs about capabilities (Self-efficacy)	
			(11) Behavioural regulation		(11) Behavioural regulation		(9) Social influences (Norms)					
							(4) Beliefs about capabilities (Self-efficacy)					
**COM-B (ultimate outcomes)**	MOTIVATION		MOTIVATION		MOTIVATION		MOTIVATION		MOTIVATION		MOTIVATION	

	Table S1: Intervention theory											
	BCT	RECOMMANDATIONS FOR IMPLEMENTATION	BCT	RECOMMANDATIONS FOR IMPLEMENTATION	BCT	RECOMMANDATIONS FOR IMPLEMENTATION	BCT	RECOMMANDATIONS FOR IMPLEMENTATION	BCT	RECOMMANDATIONS FOR IMPLEMENTATION	BCT	RECOMMANDATIONS FOR IMPLEMENTATION
	15.4		1.9		13.4		3.1		3.2		3.3	
**BEHAVIOUR CHANGE TECHNIQUES (Activities)**	**Self-talk** Prompt positive self-talk (aloud or silently) before and during the behavior	Give sporadic advice, with humor	**Commitment** Ask the person to affirm or reaffirm statements indicating commitment to change the behavior	Enable to simply validate goals and objectives by questions, only once.	**Valued self-identity** Advise the person to write or complete rating scales about a cherished value or personal strength as a means of affirming the person's identity as part of a behavior change strategy (includes 'Selfaffirmation')	Favour positive approach	**Social support (unspecified)** Advise on, arrange or provide social support (e.g. from friends, relatives, colleagues,' buddies' or staff) or noncontingent praise or reward for performance of the behavior. It includes encouragement and counselling, but only when it is directed at the behavior	Emphasize on social aspects of behaviors and the help that peers can provide	**Social support (practical)** Advise on, arrange, or provide practical help (e.g. from friends, relatives, colleagues, 'buddies' or staff) for performance of the behavior	Provide simple tips and tricks to provide practical help	**Social support (emotional)** Advise on, arrange, or provide emotional social support (e.g. from friends, relatives, colleagues, 'buddies' or staff) for performance of the behavior	Encourage a social friendlyness support without judging
**Technical recommendations**			The user can choose to use or not the option				The user can choose to use or not the option		The user can choose to use or not the option			
	↓		↓		↓		↓		↓		↓	
**THEORITICAL DOMAINS (Intermediate outcomes)**	(4) Beliefs about capabilities (Self-efficacy)		(6) Motivation and goals (Intention)		(4) Beliefs about capabilities (Self-efficacy)		(9) Social influences (Norms)		(9) Social influences (Norms)		(9) Social influences (Norms)	
	(5) Beliefs about consequences (Anticipated outcomes/attitude)				(3) Social/professional role and identity (Self-standards)		(5) Beliefs about consequences (Anticipated outcomes/attitude)		(3) Social/professional role and identity (Self-standards)			
									(11) Behavioural regulation			
	↓		↓		↓		↓		↓		↓	
**COM-B (ultimate outcomes)**	MOTIVATION		MOTIVATION		MOTIVATION		OPPORTUNITY		OPPORTUNITY		OPPORTUNITY	

	Table S1: Intervention theory							
	BCT	RECOMMANDATIONS FOR IMPLEMENTATION	BCT	RECOMMANDATIONS FOR IMPLEMENTATION	BCT	RECOMMANDATIONS FOR IMPLEMENTATION	BCT	RECOMMANDATIONS FOR IMPLEMENTATION
**BEHAVIOUR CHANGE TECHNIQUES (Activities)**	12.1		12.2		12.3		12.5	
	**Restructuring the physical environment** Change, or advise to change the physical environment in order to facilitate performance of the wanted behavior or create barriers to the unwanted behavior (other than prompts/cues, rewards and punishments)	Suggest favorable environments including creating barriers to unwanted behavior, directly in the app or by seeking social support	**Restructuring the social environment** Change, or advise to change the social environment in order to facilitate performance of the wanted behavior or create barriers to the unwanted behavior (other than prompts/cues, rewards and punishments)	Enable to join a group, favour exchanges with others	**Avoidance/ reducing exposure to cues for the behavior** Advise on how to avoid exposure to specific social and contextual/physical cues for the behavior, including changing daily or weekly routines	Raise users awareness of daily routines in order to change/improve it. Provide guidance and social support	**Adding objects to the environment** Add objects to the environment in order to facilitate performance of the behavior	Provide guidance
**Technical recommendations**								
**THEORITICAL DOMAINS (Intermediate outcomes)**	(8) Environmental context and resources (Environmental constraints)		(8) Environmental context and resources (Environmental constraints)		(8) Environmental context and resources (Environmental constraints)		(8) Environmental context and resources (Environmental constraints)	
	(11) Behavioural regulation		(9) Social influences (Norms)		(9) Social influences (Norms)		(11) Behavioural regulation	
**COM-B (ultimate outcomes)**	OPPORTUNITY		OPPORTUNITY		OPPORTUNITY		OPPORTUNITY	
