# Supplementary material for: Using theory of change to develop an intervention theory for designing and evaluating behavior change SDApps for healthy eating and physical exercise: the OCAPREV theory
Source: BMC Public Health. 2019 Nov 1;19:1435. doi: 10.1186/s12889-019-7828-4 (PMC6824130; doi:10.1186/s12889-019-7828-4)
Supplement: Supplementary file 2 — Additional file 2: Table S2. SHI criteria for SDApps. [file 12889_2019_7828_MOESM2_ESM.pdf]

Table S2: SHI Vigilance criteria for SDApps

Categories	Criteria/Components	Correspondence with BCTs included in the app (S. Michie et al., 2013)	Observability
**1/Nature of the social inequalities addressed in the app design**	It is specified that the app addresses and adapts to user characteristics, needs and circumstances.	-	Described in the store and/or in the app itself
**2/ Nature of the social inequalities addressed**	The application (app) was designed with help from a user group.	-	Described in the store and/or in the app itself
**3/Social determinant relating to social support**	The app features activities relating to social support. (Hamm et al., 2013)	3.1. Social support (unspecified)<br>3.2. Social support (practical)<br>3.3. Social support (emotional)	In the app
**4/Environmental, physical and social determinants**	The app features activities identifying resources available in the physical and social environment that may influence behavior.	4.2. Information about Antecedents<br>4.3. Re-attribution	In the app
	The app encourages users to influence their physical and social environment.	12.1. Restructuring the physical environment<br>12.2. Restructuring the social environment<br>12.5. Adding objects to the environment	In the app
**5/Economic determinants**	The app's advice and activities takes into account users' financial constraints.	-	In the app
	The app doesn't require the latest version and can work optimally on multiple versions, not just on the most recent ones.	-	In the app
	There is no need to pay for additional modules for the app to work properly.	-	In the app

	The app is free or inexpensive	-	In the app
	The app makes it easier for vulnerable groups to access incentives (gifts, discount vouchers…);	-	Described in the store and/or in the app itself
**6/Transparency with regard to commercialization, influence /Quality**	Designers and financial backers are clearly identified and specified.	-	Described in the store and/or in the app itself
	The app features validated content, or refers to evidence-based recommendations or methods.	-	In the app
	The roles and input of third parties (healthcare professionals, coaches, peers…) who may intervene in the app in combination with it are clearly defined.	-	Described in the store and/or in the app itself
**7/Adaptation of content to differences in representation / expectations**	The app features activities that help personal goal setting. (self-normativity).	1.1. Goal setting (behavior)<br>1.5. Review behavior goal(s)	In the app
	The app's content draws on the users' own habits.	-	In the app
	The activities and information featured are adapted to people's needs and expectations: based on a questionnaire or on the freedom to do or not to do an activity.	-	In the app
**8/Adaptation to user literacy**	The language used is easy to understand by everyone (Latulippe et al., 2017)	-	In the app
	Navigation is user-friendly (Latulippe et al., 2017)	-	In the app
	The apps has no more than 8 reading grade levels 5)	-	In the app
	Navigation is easy (Berland et al., 2001)	-	In the app
	Increased use of audio and video (Latulippe et al., 2017)	-	In the app
	The apps focuses on activities that are both fun and educational activities (Latulippe et al., 2017)	-	In the app
**9/Accessibility**	If the app features activities, these take into account various levels of mobility or disability.	-	In the app

	Access to the app is universal 24): it doesn't need an internet connection all the time.	-	In the app
	The content doesn't stigmatize.	-	In the app
	App content adapts to religious differences.	-	In the app
	App content respects users' cultural attributes (Latulippe et al., 2017): values, languages, adapted images, diverse testimonials, relating to different cultures.	-	In the app
**10/Ability to empower**	The apps features activities aimed at empowering users.	All the intervention theory's BCTs strengthen "capability"	In the app
	The app leans toward activities that are adapted to the needs expressed.	-	In the app
	The app features activities for improving users' technical skills (know-how, abilities) above all in that they:<br><br>• allow capabilities to be developed<br>• advise on how to implement change<br>• allow objectives to be set or reset to be adapted to needs<br>• allow users to analyze themselves and to define what motivates them or not.	All the intervention theory's BCTs strengthen "capability". And in particular:<br>1. Goals and planning (including 1.2. Problem solving (decisional balance))<br>2. Feedback and monitoring	In the app
	The app features activities for developing self-esteem	15.1 Verbal persuasion about capability<br>15.3 Focus on past success<br>15.4 Self-talk	

	The app features activities for users to develop critical awareness: identifying the causes of a problem to solve, understanding that the causes and solutions of the problem experienced do not fully come under the user's responsibility etc.	1.2. Problem solving<br>4.2. Information about Antecedents<br>4.3. Re-attribution<br>4.4 behavioral experiment<br>9.2. Pros and cons	
